# Supplementary material for: The promise of long-read RNA-seq: reducing bias in analyses of allele imbalance
Source: NAR Genom Bioinform. 2026 Aug 4;8(3):lqag071. doi: 10.1093/nargab/lqag071 (PMC13434171; doi:10.1093/nargab/lqag071)
Supplement: Supplementary file 1 [file lqag071_supplemental_files.zip › supp-3.docx]

**Supplementary Table 1:** Overview of studies using long-read RNA-seq for quantification of allele-specific expression analysis.

|  | **Study** | **Species** | **Reference input** | **Mapping strategy** | **Assignment of reads to haplotypes** |
| --- | --- | --- | --- | --- | --- |
| **Sequential haplotype mapping** | (Glinos et al., 2022) | human | Diploid genome, long-read RNA-seq was used to correct haplotypes | Mapping to haplotypes **sequentially** | Counting the number of read containing REF or ATL allele |
|  | (Zheng et al., 2022) | Hexaploid bamboo | Reference genomes from parents | Aligning to parental genomes with minimap2 **sequentially** | Assignment of reads based on SNPs |
|  | (Feng et al., 2021) | Hybrid rice | Reference genomes from both parents | Aligning to parental genomes with minimap2 **sequentially** | Assignment of reads based on SNPs |
| **Competitive genotype mapping** | (Hughes et al., 2023) | Human, HLA | Human reference genome, screening HLA transcript database with long-reads and generation of reference with allele-specific HLA types. | Aligning to HLA type personalized reference genome **competitively** with minimap2 | Assignment based on primary minimap2 mapping. |
|  | (Bresnahan et al., 2023) | reciprocal honeybee crosses | combined parental transcriptomes | Alignment with minimap2 **competitively** to transcriptomes | Not clear: potentially assignment based on primary minimap2 mapping. |
|  | (Dominguez-Ortiz et al., 2024) | human ovarian cancer | annotated Gencode. v44 BRCA1 transcripts | alignment with salmon with long-read parameters **competitively** | Not clear: potentially assignment based on primary mapping. |
|  | (Cornaby et al., 2022) | human HLA | Human reference genome, screening HLA transcript database with long-reads and generation of reference with allele-specific HLA types. | Aligning to HLA type personalized reference genome **competitively** with minimap2 | Assignment based on primary minimap2 mapping. |
| **Mapping to a single haplotype** | (Park & Cenik, 2024) | human and mouse | **N-masked** reference genome | alignment with minimap2 to reference transcriptome | Assignment based on known SNPs |
|  | (S.-Y. Chen, 2022) | F1 hybrids of cattle and yak | **N-masked** reference genome | Alignment with minimap2 | Assignment based on distinguishable interspecies SNPs |
|  | (Xu et al., 2024) | Hybrid maize (B73 × Ki11, Ki11 × B73) | maize RefGen_v4 | Aligning to one parent with minimap2 | **Phasing** of long-read RNA-seq reads over SNP positions with IsoPhase (Wang et al., 2020) |
|  | (Low et al., 2020) | Angus and Brahman crossbred cattle | Reference genome from one parent | Aligning to one haplotype with minimap2 | **Phasing** of long-read RNA-seq reads over SNP positions with IsoPhase (Wang et al., 2020) |
|  | (Wang et al., 2020) | Hybrid maize (B73 × Ki11, Ki11 × B73) | Reference genome from one parent | Aligning to one haplotype with minimap2 | **Phasing** of long-read RNA-seq reads over SNP positions with IsoPhase (Wang et al., 2020) |

**References**

Bresnahan, S. T., Lee, E., Clark, L., Ma, R., Rangel, J., Grozinger, C. M., & Li-Byarlay, H. (2023). Examining parent-of-origin effects on transcription and RNA methylation in mediating aggressive behavior in honey bees (Apis mellifera). BMC Genomics, 24(1), 315. https://doi.org/10.1186/s12864-023-09411-4

Chen, S.-Y. (2022). Favoring Expression of Yak Alleles in Interspecies F1 Hybrids of Cattle and Yak Under High-Altitude Environments. Frontiers in Veterinary Science, 9.

Cornaby, C., Montgomery, M. C., Liu, C., & Weimer, E. T. (2022). Unique Molecular Identifier-Based High-Resolution HLA Typing and Transcript Quantitation Using Long-Read Sequencing. Frontiers in Genetics, 13, 901377. https://doi.org/10.3389/fgene.2022.901377

Dominguez-Ortiz, J., Álvarez-Gómez, R. M., Montiel-Manríquez, R., Cedro-Tanda, A., Alcaraz, N., Castro-Hernández, C., Bautista-Hinojosa, L., Contreras-Espinosa, L., Torres-Maldonado, L., Fragoso-Ontiveros, V., Sánchez-Contreras, Y., González-Barrios, R., Fuente-Hernández, M. A. D. la, Mejía-Aguayo, M. de la L., Juárez-Figueroa, U., Padua-Bracho, A., Sosa-León, R., Obregon-Serrano, G., Vidal-Millán, S., … Herrera-Montalvo, L. A. (2024). A Molecular Characterization of the Allelic Expression of the BRCA1 Founder Δ9-12 Pathogenic Variant and Its Potential Clinical Relevance in Hereditary Cancer. International Journal of Molecular Sciences, 25(12), 6773. https://doi.org/10.3390/ijms25126773

Feng, J.-W., Lu, Y., Shao, L., Zhang, J., Li, H., & Chen, L.-L. (2021). Phasing analysis of the transcriptome and epigenome in a rice hybrid reveals the inheritance and difference in DNA methylation and allelic transcription regulation. Plant Communications, 2(4), 100185. https://doi.org/10.1016/j.xplc.2021.100185

Glinos, D. A., Garborcauskas, G., Hoffman, P., Ehsan, N., Jiang, L., Gokden, A., Dai, X., Aguet, F., Brown, K. L., Garimella, K., Bowers, T., Costello, M., Ardlie, K., Jian, R., Tucker, N. R., Ellinor, P. T., Harrington, E. D., Tang, H., Snyder, M., … Cummings, B. B. (2022). Transcriptome variation in human tissues revealed by long-read sequencing. Nature, 608(7922), 353–359. https://doi.org/10.1038/s41586-022-05035-y

Hughes, A. E. O., Montgomery, M. C., Liu, C., & Weimer, E. T. (2020). Quantification of Allele-Specific HLA Expression with Nanopore Long-Read Sequencing. Blood, 136(Supplement 1), 42–43. https://doi.org/10.1182/blood-2020-140902

Hughes, A. E. O., Montgomery, M. C., Liu, C., & Weimer, E. T. (2023). Allele-specific quantification of human leukocyte antigen transcript isoforms by nanopore sequencing. Frontiers in Immunology, 14, 1199618. https://doi.org/10.3389/fimmu.2023.1199618

Low, W. Y., Tearle, R., Liu, R., Koren, S., Rhie, A., Bickhart, D. M., Rosen, B. D., Kronenberg, Z. N., Kingan, S. B., Tseng, E., Thibaud-Nissen, F., Martin, F. J., Billis, K., Ghurye, J., Hastie, A. R., Lee, J., Pang, A. W. C., Heaton, M. P., Phillippy, A. M., … Williams, J. L. (2020). Haplotype-resolved genomes provide insights into structural variation and gene content in Angus and Brahman cattle. Nature Communications, 11(1), 2071. https://doi.org/10.1038/s41467-020-15848-y

Park, D., & Cenik, C. (2024). Long-read RNA sequencing reveals allele-specific N 6 -methyladenosine modifications. https://doi.org/10.1101/2024.07.08.602538

Wang, B., Tseng, E., Baybayan, P., Eng, K., Regulski, M., Jiao, Y., Wang, L., Olson, A., Chougule, K., Buren, P. V., & Ware, D. (2020). Variant phasing and haplotypic expression from long-read sequencing in maize. Communications Biology, 3(1), 78. https://doi.org/10.1038/s42003-020-0805-8

Xu, F., Liu, S., Zhao, A., Shang, M., Wang, Q., Jiang, S., Cheng, Q., Chen, X., Zhai, X., Zhang, J., Wang, X., & Yan, J. (2024). iFLAS: Positive-unlabeled learning facilitates full-length transcriptome-based identification and functional exploration of alternatively spliced isoforms in maize. The New Phytologist, 241(6), 2606–2620. https://doi.org/10.1111/nph.19554

Zheng, Y., Yang, D., Rong, J., Chen, L., Zhu, Q., He, T., Chen, L., Ye, J., Fan, L., Gao, Y., Zhang, H., & Gu, L. (2022). Allele-aware chromosome-scale assembly of the allopolyploid genome of hexaploid Ma bamboo (Dendrocalamus latiflorus Munro). Journal of Integrative Plant Biology, 64(3), 649–670. https://doi.org/10.1111/jipb.13217
